# The ICU Care Plan: Human-Centered Design of a Tool to Support Time-Limited Trials for Older Adults With Critical Illness

**DOI:** 10.1111/jgs.70155

**Published:** 2025-10-13

**Authors:** Sean M. Mortenson, Josephine M. McCartney, Joy X. Moy, Geralyn M. Palmer, Jaime H. Goldberg, Demetrius B. Solomon, Madison Polley, Neera Grover, Margaret L. Schwarze, Toby C. Campbell, Jane L. Holl, Sarah L. Esmond, Jacqueline M. Kruser

**Affiliations:** 1 Department of Medicine, Division of Allergy, Pulmonary and Critical Care Medicine, University of Wisconsin School of Medicine and Public Health, Madison, Wisconsin, USA; 2 Hackensack Meridian School of Medicine, Clifton, New Jersey, USA; 3 Department of Medicine, University of Wisconsin School of Medicine and Public Health, Madison, Wisconsin, USA; 4 Sandra Rosenbaum School of Social Work, University of Wisconsin, Madison, Wisconsin, USA; 5 Department of Industrial and Systems Engineering, University of Wisconsin, Madison, Wisconsin, USA; 6 Department of Surgery, Division of Vascular Surgery, University of Wisconsin School of Medicine and Public Health, Madison, Wisconsin, USA; 7 Division of Hematology, Medical Oncology and Palliative Care, University of Wisconsin, Madison, Wisconsin, USA; 8 Department of Neurology, The University of Chicago, Chicago, Illinois, USA; 9 Institute for Clinical and Translational Research, University of Wisconsin, Madison, Wisconsin, USA

**Keywords:** critical care, design thinking, palliative care, time-limited trial

## Abstract

**Background:**

For older adults with critical illness, decisions about life-sustaining therapies can be challenging. A time-limited trial (TLT) is a collaborative care plan endorsed by experts in palliative and critical care to help navigate these challenges. TLTs entail trying life-sustaining therapy for a defined duration. Response to treatment then informs whether to continue recovery-directed care or shift focus exclusively to comfort. TLTs require collaboration among clinicians, patients, and/or surrogate decision makers, yet there is little practical guidance on how to accomplish this. Thus, we sought to design a collaborative TLT planning tool and characterize its valued characteristics.

**Methods:**

In this qualitative study framed by human-centered Design Thinking, we conducted a series of semi-structured interviews (*n* = 25) and focus groups (*n* = 5) with 28 participants who were (1) older adults (age ≥ 65) with serious illness, (2) adults of any age with surrogate decision-making experience for an older adult, and/or (3) intensive care unit (ICU) physicians. We purposively sampled across a Midwestern state to achieve diverse representation and used the Rigorous and Accelerated Data Reduction (RADaR) technique for qualitative analysis.

**Results:**

We used participants’ input to design the *ICU Care Plan*, a paper-based tool consisting of a fillable template. The tool is designed to guide a collaborative TLT planning conversation among clinicians, patients, and surrogates and then serve as a visual summary of the care plan. Participants endorsed the tool as (1) creating a unified frame of reference for a complex process; (2) promoting transparency; and (3) setting and managing expectations. The tool exemplifies participants’ design priorities of simplicity and flexibility.

**Conclusions:**

We used a human-centered design process to develop a tool for in-the-moment TLT planning that is endorsed by older adults, surrogates, and ICU physicians. Low technology, intentionally simple interventions are a promising approach to promote patient- and family-centered collaboration.

## 1 | Introduction

For older adults with critical illness, choices about whether to use life-sustaining therapies (LST) are often framed as a dichotomy: a choice between LST without boundaries or immediate comfort-focused, end-of-life care. However, many older adults prefer a more nuanced approach, extending life with LST when possible while avoiding its prolonged use if the chance of survival or of an acceptable quality of life becomes too low [1–6]. A time-limited trial (TLT) is an approach to patient care proposed by experts in palliative and critical care, to support this middle-ground set of priorities and to help navigate the challenges of these high stakes decisions [7]. A TLT is a collaborative agreement between patients, their surrogate decision makers (herein surrogates), and clinicians to use life-sustaining treatment for a defined duration [8]. The patient’s response to therapy during this “trial” informs the decision of whether to continue recovery-focused care or transition to care focused exclusively on comfort near the end of life.

Fundamentally, a TLT is a collaborative process among clinicians, patients, and/or their surrogates [8, 9]. Yet, patient and surrogate perspectives about how best to accomplish TLT planning have never been examined. As a result, little is known about how clinicians should engage with patients and surrogates to successfully plan and reach agreement about a TLT. This planning process can be challenging and fraught, especially given the stressful, in-the-moment context of acute critical illness. Indeed, observational studies demonstrate substantial variation in how clinicians currently discuss TLTs with patients and surrogates and in how such care plans are received and understood [4, 10].

Design Thinking is a problem-solving approach rooted in the fields of engineering, computer science, psychology, and business. This cross-disciplinary approach is defined by its intentional integration of end-users (e.g., patients, surrogates) in the process of developing solutions for complex problems [11]. This human-centered, ground-up approach has become an increasingly common strategy in healthcare delivery science to improve the impact, implementation, and sustainability of new interventions [12]. Given the promise of Design Thinking to generate effective solutions for complex problems, we sought to apply this framework in the development of a novel tool to help clinicians, older adults, and/or their surrogates collaboratively plan a TLT in the context of critical illness. The objective of this article is to present the tool resulting from this design process and the findings from our qualitative analysis characterizing the tool and key design features that guided its development.

## 2 | Methods

### 2.1 | Study Design

**FIGURE 1 |:**
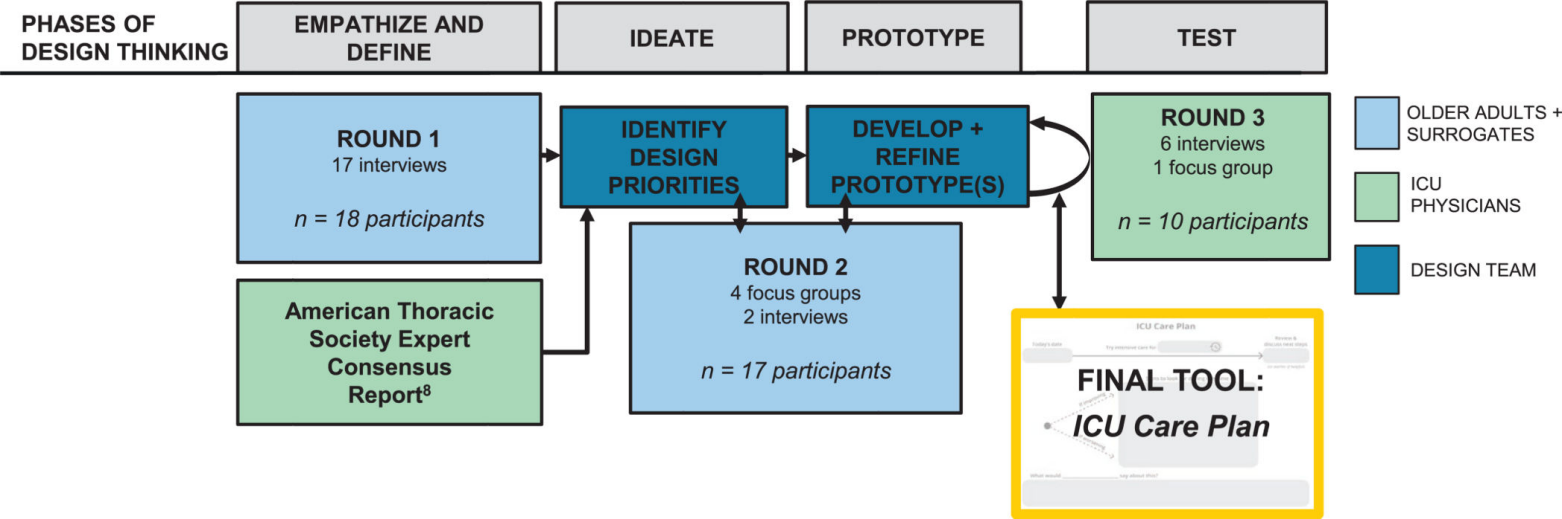
The five-phase Design Thinking process to develop the ICU Care Plan tool. We conducted 30 study sessions (focus groups and interviews) across 3 rounds of data collection to develop the *ICU Care Plan*. In Round 1, we conducted individual interviews to allow privacy for participants to share their personal perspectives and stories. A total of 17 interviews were conducted with 18 participants because 2 participants (an older adult and their surrogate) preferred to participate together. In Round 2, we offered either focus groups or interviews according to participant preference. Of the 18 Round 1 participants, 17 were retained in Round 2 of the study. In Round 3, we offered either interviews or focus groups according to ICU physician preference and scheduling availability.

We conducted a qualitative study consisting of multiple semi-structured interviews and focus groups, organized by the iterative, five-phase process of Design Thinking (Figure 1): empathize (understand users’ needs), define (clearly state the problems, as defined by users), ideate (generate ideas for multiple potential solutions), prototype (build multiple, low-resource prototypes of solutions), and test (test prototypes with users). The study was reviewed and approved by the University of Wisconsin Institutional Review Board, and all study participants provided informed consent. This article follows the Standards for Reporting Qualitative Research [13].

### 2.2 | Participants

We recruited individuals representative of ICU patients and/or surrogates: older adults (≥ 65 years) with serious illness, defined as ≥ 1 of the following diagnoses: cancer, heart failure, chronic lung disease, kidney disease with dialysis, cirrhosis, diabetes with severe complications; or ≥ 2 hospitalizations in the preceding year [14] and/or adults of any age (≥ 18 years) with experience making a major medical decision on behalf of an older adult. During the test phase of the design process, we also recruited practicing ICU physicians. We excluded those who could not participate in the study activities in English.

Recruitment was conducted in a single Midwestern state in the United States and was supported by a partnership with three regional Area Health Education Centers (AHECs) [15–17]. For older adults and surrogates, we distributed recruitment emails and flyers through the AHECs and their local community organization partners. Among eligible respondents, we used purposive, maximum variation sampling [18] to ensure the inclusion of individuals with varied racial and ethnic identities and from both rural and urban areas. For older adults/surrogates, we continued sampling until reaching code saturation [19] in the first round of study sessions (see below for Round 1 details), defined as the point at which no additional needs for TLT planning were identified with additional participants. For ICU physicians, we used snowball sampling [18] starting with the study teams’ professional networks to recruit via professional email addresses, purposively sampling to achieve variation in practice location. We defined saturation within the physician sample as the point at which no new perspectives on or refinements for the tool were identified with additional participants.

### 2.3 | Design Thinking Process

We conducted a total of 30 study sessions, which were organized by the 5 Design Thinking phases (Figure 1). Sessions were conducted using the qualitative methods of semi-structured interviews (*n* = 25) and focus groups (*n* = 5). To support recruitment and retention across study sessions, we offered both in-person and video conference (Zoom Video Communications Inc., San Jose, CA) options and provided a gift card incentive following each session. Sessions were audio-recorded, then transcribed and de-identified by a professional, secure service for analysis. Interviews and focus groups were conducted by two experienced qualitative researchers with backgrounds in medicine (J.X.M.) and social work (J.H.G.). The session guides (Texts S1, S2, S3) were developed by investigators based on Design Thinking phase goals, with input and revisions from the project advisory board, which included AHEC community partners, a prior ICU surrogate, and those with professional expertise in communication, health equity, and user-centered design.

During Round 1 study sessions (*empathize and define*), we introduced older adults/surrogates to the concept of a TLT using a story board of a hypothetical patient case (Text S4). In these empathize and define sessions, we developed a shared understanding about TLTs and elicited older adults/surrogates’ needs for TLT planning. We used the Rigorous and Accelerated Data Reduction (RADaR) [20] technique to efficiently organize Round 1 data and conduct initial qualitative analyses on session transcripts. A team of three investigators from different backgrounds (social work and palliative care [J.H.G.]; systems engineering [D.B.S.]; medicine [J.M.M.]) reduced the Round 1 transcripts to data tables pertaining to TLTs and the empathize, define, and ideate Design Thinking goals. These investigators then conducted the first round of RADaR coding, using a deductive approach to identify major themes specific to tool development.

Using the Round 1 RADaR analysis results, a five-person design team (J.M.K., T.C.C., M.L.S., J.L.H., N.G.) with diverse expertise and experience (palliative and critical care, ethics, communication, user-centered design research, prior ICU surrogate) identified key design targets, worked to *ideate* potential solutions, and then developed initial *prototypes*. In developing prototypes, the design team also drew from a consensus report from the American Thoracic Society on the essential elements of TLTs [8] and from their prior work developing communication tools [21, 22].

In Round 2 sessions with the same older adults/surrogates (*ideate and prototype*), we used visual storyboarding of the same Round 1 hypothetical patient and provided participants with multiple tool prototypes to gather perspectives, refine components, and ideate about potential applications. Following the same RADaR process described above, the design team iteratively refined the tool during and after Round 2. After converging on a single prototype based on older adult/surrogate input, we conducted Round 3 (*test*) interviews and focus groups with ICU physicians to elicit their perspectives. Physician participants were also asked to simulate using the tool with the surrogate of a hypothetical patient.

### 2.4 | Integrated Qualitative Analysis

Following completion of all 3 rounds of data collection, we conducted a second RADaR process with another team of investigators (critical care [S.M.M., G.M.P., J.M.K.], medicine [J.X.M., J.M.M.]). The purpose of this second analysis was to integrate data from all participants/sessions to formulate higher-level themes. During both processes, at least two coders independently reviewed the same RADaR data tables, labeling sections of text with descriptive codes. The entire coding team then met regularly during the coding process to compare codes, build the coding taxonomies, refine codes and definitions, and review and agree on consensus code(s) for sections of text. As coding was nearing completion, the team meetings also involved developing higher-level themes to describe relationships and patterns within and between codes.

## 3 | Results

**FIGURE 2 |:**
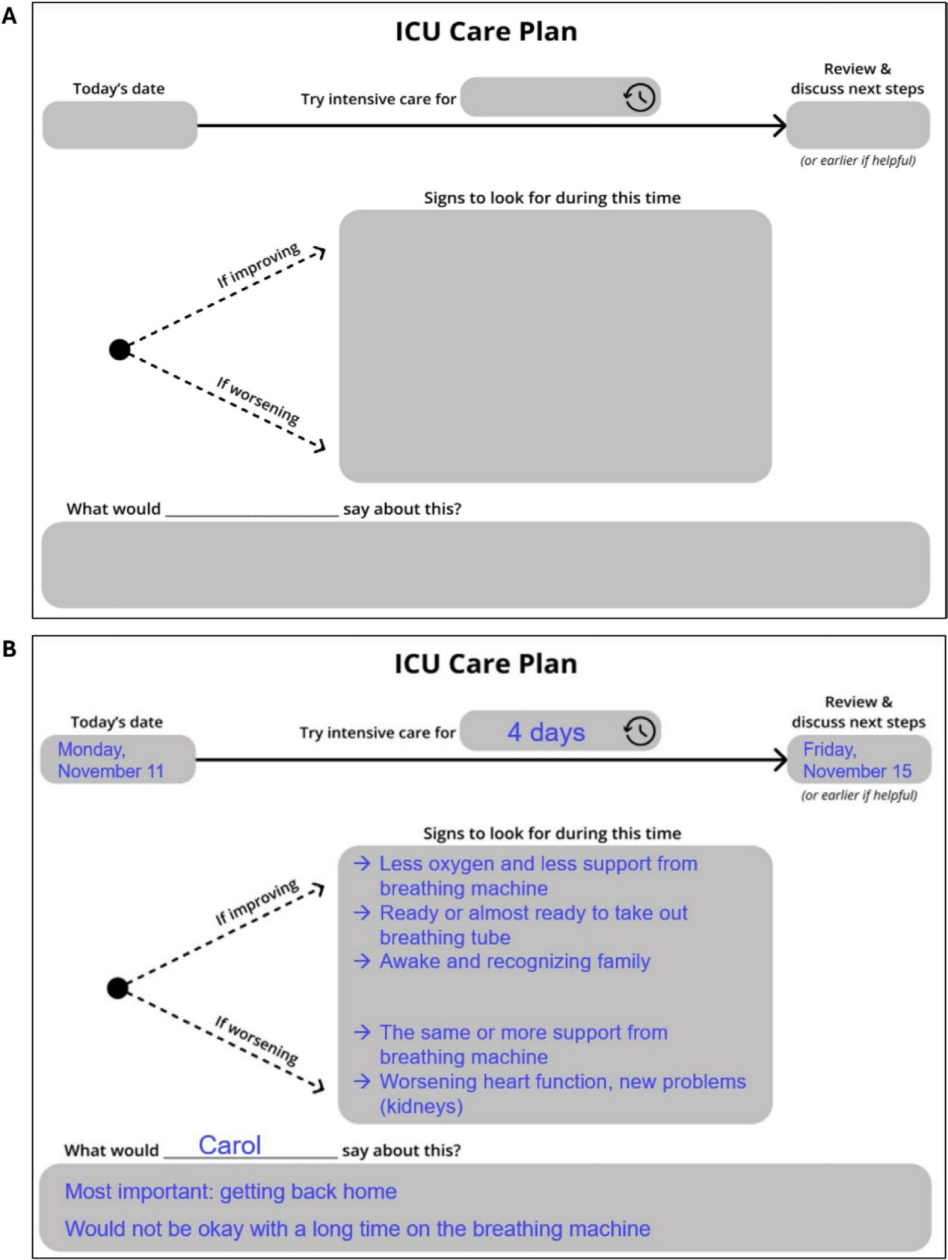
The ICU Care Plan. (A) This paper, fillable template is a tool to guide and serve as a summary of a time-limited trial planning conversation among clinicians, patients, and/or their surrogate decision makers and family members. (B) Example of completed template using details from a hypothetical patient case.

A total of 28 participants took part in the study (Table 1). The 30-session, 5-phase design process culminated in a final tool called the *ICU Care Plan* (Figure 2). Examples of the iterative prototypes, highlighting the design evolution throughout the process, are shown in Figure S1. The *ICU Care Plan* is a paper-based, fillable template that visually depicts the major components of a TLT. The tool is designed to facilitate a TLT planning conversation between ICU clinicians and patients and/or surrogates. Together, through collaborative discussion and deliberation, they are expected to complete the template. A typical setting for using the tool is an ICU family meeting. Following the conversation, the tool also serves as a tangible, visual summary of the TLT plan, which can be referenced in future conversations and shared among family members and the clinical team.

### 3.1 | Valued Characteristics of the ICU Care Plan

When reviewing and refining *ICU Care Plan* tool prototypes in Round 2, all older adult and surrogate participants endorsed the general tool design. In the Round 3 test sessions with ICU physicians, all participants reported that they would use the tool in their clinical practice, though one voiced a desire to wait for evidence about its impact on patients and families before adoption.

We identified several tool characteristics that were highly valued by participants (Table 2). First, the tool creates a single, shared frame of reference for an inherently complex process. It was described as “unifying,” which is especially important for TLTs that unfold over days, involve multiple individuals, and often require sensitive, difficult conversations about the end of life. The *ICU Care Plan* was seen as a central reference point to unify all individuals involved in a TLT, including the patient, their family and other loved ones, and their clinical teams. Participants noted that this shared frame of reference could even help unify the continuously rotating clinical team members in the ICU:

Let’s say a new intern comes in, or a resident comes in, they can look at that and see what the family or what the ICU has already put in place so that they don’t go changing things.

(Older adult, participant 11)

Participants further noted that the *ICU Care Plan* could support the follow-through of a planned TLT:

I want to get this in writing in a way so that we all have a shared frame of reference that we can look back to. And the reason we’re going to use this is, is in about two or three days […] we’re going to regroup and look back at this and say, where are we, relative to what we wrote down?

(ICU physician, participant 25)

Older adults and surrogate participants viewed the tool as promoting transparency, by specifically illustrating the uncertainty in the patient’s outcome and the potential for deterioration, including the possibility of death. Participants also valued the tool as a concrete plan in the face of this uncertainty, comprised of “incremental” steps toward a future course of action. Participants characterized this transparency as both promoting trust and meeting their expectations for honesty from clinicians:

I almost feel that in this case, the doctor gained that trust, because it was very clear from the beginning. We’re going to try this, this is the timeframe, these are the days that we’re going to try. And if this doesn’t work, then we need to follow on this. The clarity and transparency of that communication really put it like, I’m here to be honest with you, and I’m not hiding anything from you.

(Surrogate, participant 15)

Participants valued the tool because it helped to establish clear expectations for an ICU stay, even in the setting of uncertainty. One participant compared the *ICU Care Plan* to a map used while hiking in an unfamiliar area:

When you set out, you don’t always know the destination you’re going, but you kind of have a map, you know how to get back home in some ways. It is a tool; it is an aid.

(Surrogate, participant 10)

Despite these endorsements, we also identified important considerations for using the tool in clinical practice. The tool is not designed as a stand-alone intervention. Participants emphasized that the *ICU Care Plan* should be used alongside and implemented through communication best practices. Furthermore, the purpose of the graphic aid is to serve as an outline and high-level summary for a conversation that requires substantial, additional dialog and detail. Multiple participants highlighted the importance of considering prior advance care planning efforts while using the tool and discussing a TLT. Several ICU physicians raised a concern that some patients and families would not be good candidates for the tool, though we were unable to identify any themes to further characterize this concern.

### 3.2 | Key Themes That Guided Tool Development

Participants preferred a paper format for the *ICU Care Plan*, which was described as “grounding” and “tangible” by participants. This informed our design choice to develop a paper tool. Paper was described as more universally transferable and accessible compared to an electronic format: “in general, everyone can handle a piece of paper, as opposed to people being able to get online” (Older adult, participant 3). A paper format was seen as especially important for older adults, who “are not always thrilled with electronic anything” (Surrogate, participant 9).

Participants also endorsed the relatively durable nature of a paper tool, which could be kept as a reference and reminder of what was said and planned:

The thing most helpful would be having a visual that you can reference back to, because in a time when you’re dealing with a loved one going through things, you’re more caught in your emotions. […] you won’t remember those things, […] and people may have different interpretations of what was said. But when you have an actual visual in a plan, I think that’s something to be able to reference back to.

(Surrogate, participant 18)

The durability of a paper-based tool also affords an opportunity to share the tool and its contents with others. Participants suggested multiple methods of sharing, including making copies, hanging a copy in the patient’s hospital room for clinicians and family, scanning into the electronic health record, and photographing to share through text or email. ICU physicians noted the possibility of using this tool to improve patient handoffs between ICU clinicians who are continually rotating.

Despite general endorsement of a paper-based tool, one ICU physician raised a concern about how a physical document might constrain a conversation occurring among multiple people. The physician said:

It’s an 8.5 by 11 sheet of paper. So, I think the trouble you could run into with a document this small is […] if you’ve got four people who are making decisions, and only one person can see that piece of paper at a time […]. It might well be that you have the whole conversation and then you say, I’m going to summarize what we just talked about, and scribble this down on this piece of paper and hand it over and say, […] Okay, I’m going to copy this and give it to you so that you all have this as a touchstone.

(ICU physician, participant 25)

This observation informed the development of the tool as a high-level summary and visual representation of a conversation taking place between clinicians and patients/surrogates, but not as an intervention that supplants or comprehensively documents that conversation.

In all sessions, participants prioritized two major design features of the tool: simplicity and flexibility (Table 3). Simplicity was valued because it promotes understanding, especially in the stressful context of critical illness:

I think it would keep it simple for non-medical persons […] it’s simple. It’s right there. Oh, [the patient] this is her plan, and this is what we’re looking for, what they’re hoping for, any kind of concerns. It’s laid out, it’s laid out simple.

(Surrogate, participant 19)

Participants advocated for simplicity as a way to ensure all individuals (e.g., family, clinicians) involved in a patient’s care shared the same understanding of the care plan, reinforcing the shared frame of reference theme. Participants also advocated for simple, unambiguous, and clear language. For example, an early prototype had the title, *Our ICU Plan*. This language was intended to be inclusive and collaborative, but participants found it confusing and ambiguous: “it’s a matter of a question of [the word] ‘our’ […]. I think it has to be somewhat more specific. And the medical team and the patient’s family may have very different points of view here” (Older adult, participant 13). Thus, we retitled the tool *ICU Care Plan*.

Participants identified flexibility as a high priority for the tool. This flexibility was described in two dimensions: (1) to individualize the template for each patient and (2) to convey that the care plan was not binding or overly rigid. Both ICU physician and older adult/surrogate participants wanted a tool that could be individualized to account for each patient’s unique clinical context and their personal goals and priorities. This led the design team to use general phrases (e.g., “try intensive care”) instead of more specific terms (e.g., life support). Intentional white spaces were added to the tool for clinicians to insert patient-specific, individualized information when necessary. The patient-voice section of the template was also intentionally placed, spanning the entire care plan timeline, “as overarching things that kind of ground us; having those listed at the bottom helps communicate that those are all around” (Surrogate, participant 10). To convey flexibility in the care plan, the design team intentionally selected words and phrases that imply iteration and flexibility (try, if, discuss, review) and visual signals (dotted instead of solid lines).

## 4 | Discussion

Using a human-centered Design Thinking approach, we developed a novel tool to support the collaborative planning of a TLT. The resulting *ICU Care Plan* is a paper-based, fillable template that guides a TLT planning conversation between clinicians and a patient with critical illness and/or their family and then serves as a visual, tangible summary of this conversation. The tool was unanimously endorsed by study participants as creating a shared and unified frame of reference, promoting transparency, and setting and managing expectations. The *ICU Care Plan* exemplifies the design features of simplicity and flexibility, which were prioritized by study participants.

Although TLTs are increasingly discussed in the healthcare literature and used in clinical practice, there are very few tools and interventions available to support and promote their use. As a result, observational studies demonstrate wide variation in current use of TLTs [4, 10]. Prior work seeking to improve the use of TLTs has focused on expert-based communication guidance and patient- and family-facing educational materials about the general TLT concept [9, 23]. Our study advances this work by introducing a novel clinical tool that supports point-of-care TLT planning for older adults facing acute critical illness. Using a human-centered, ground-up approach, we developed an intentionally simple, flexible, and tangible tool that was described as unifying and transparent by patients and surrogates. These tool characteristics are crucial for the challenging process of engaging patients and surrogates in high-stakes decisions about life-sustaining therapy—which entail discussing difficult concepts such as uncertainty, the possibility of deterioration and death, and the limits of medical care. An important next step will be to conduct a clinical trial to test whether the *ICU Care Plan* improves patient, family, and/or clinician outcomes compared to current practice.

Our findings have important implications for others who seek to improve communication and collaboration with older adults, beyond the specific context of time-limited trials. In an era where electronic, online, app-based interventions are the dominant approach, it is notable that our human-centered design approach led to a low-technology, paper-based, intentionally simple tool. The rationale for this low-technology design was not just to address the barriers of technological literacy and access. We found that older adults, surrogates, and ICU physicians perceived a paper, physical artifact of a conversation as more tangible and grounding—which may be important attributes when promoting communication about emotional, high-stakes topics. The preference for a paper-based tool also aligns with prior work in the similar context of high-stakes surgical communication [22, 24]. One practical benefit of this design is the ease of multi-modal dissemination tailored to the unique ICU environment—making copies, hanging in patient rooms, scanning into the EHR, taking a picture with cell phones to text or email it. These findings align with and support the growing interest in “frugal innovation” across healthcare. Frugal innovation stands in contrast to the traditional focus in medicine on high-technology, complex innovations that require substantial resources and infrastructure [25]. Frugal innovation favors intentional simplicity and focuses on core functions of an innovation instead of embellishment and multiple features [26], leading to low-cost, creative, context-tailored solutions that can be easily scaled. This type of innovation has also taken hold in manufacturing, food, and other industries [27], and our study supports its promise in healthcare.

When interpreting the results, some limitations should be considered. The study was conducted in English and in a single Midwestern state, so the priorities and perspectives of our sample might not be transferable to other regions. However, we did use purposive sampling to successfully gather participants with varied lived experiences to support inclusive design. Participants were also community-dwelling older adults and caregivers, so their perspectives might differ from those who are using the tool in the moment of an acute, critical illness. We also relied on low-fidelity simulation testing with ICU physicians (e.g., walk-through of a hypothetical case and conversation), so their real-world use of the tool may differ from our evaluation setting. Future testing of this tool in real-time clinical practice will be important for further refinement and evidence of effectiveness.

We used Design Thinking to develop a paper-based, fillable template tool that supports in-the-moment TLT planning for older adults with critical illness. The *ICU Care Plan* tool is endorsed by older adults, surrogates, and ICU physicians as being unifying and transparent. Our findings suggest that low-technology interventions that feature intentional simplicity and flexibility are a promising approach to promote communication and collaboration among clinicians, older adults, and their surrogates.

## Supplementary Material

Supporting Information

Additional supporting information can be found online in the Supporting Information section. Text S1: Round 1 interviewer/moderator guide. Text S2: Round 2 interviewer/moderator guide. Text S3: Round 3 interviewer/moderator guide. Text S4: Hypothetical patient case used to introduce participants to the concept of a time-limited trial. Figure S1: Examples of iterative prototypes developed and refined throughout the Design Thinking process.

## Figures and Tables

**TABLE 1 |:** Demographics and characteristics of study participants.

Older adult and surrogate participants	* n* = 18^a^

Age, *n* (%) < 65 years	7 (38.9)
≥ 65 years	11 (61.1)
Female, *n* (%)	16 (88.9)
Race and ethnicity, *n* (%) Asian	1 (5.6)
Black or African American	3 (16.6)
Hispanic/Latinx	4 (22.2)
White or Caucasian	10 (55.6)
Prior healthcare experiences^b^, *n* (%) Personal experience with major medical condition/s	6 (33.3)
Surrogate decision maker	15 (83.3)
Previous ICU experience (as patient or surrogate)	14 (77.8)
Residential location^c^, *n* (%) Rural^d^	5 (27.8)
Urban^e^	13 (72.2)

**ICU physician participants**	***n* = 10**

Years since completion of critical care training, (*n* %) 0–5	1 (10.0)
6–10	3 (30.0)
11–20	5 (50.0)
21–30	1 (10.0)
Female, *n* (%)	2 (20.0)
Race and ethnicity, *n* (%) Asian^f^	3 (30.0)
Black or African American	1 (10.0)
Hispanic/Latinx	0 (0.0)
White	5 (50.0)
Other^g^	1 (20.0)
Location of practice Urban	10 (100.0)
Rural	0 (0.0)
Regularly take care of patients from rural areas^h^	8 (80.0)

a 1 participant did not complete the 2nd interview/focus group (female, < age 65, caregiver, Hispanic).

b Self-identified on screening.

c Based on zipcode provided by participant; categorized using primary RUCA Codes, 2010.

d Includes RUCA codes for small town and rural.

e Includes RUCA codes for metropolitan and micropolitan.

f Includes 1 “other” self-report as South Asian.

g “Other” with no additional response (1); 0 participants identified as American Indian or Alaska Native; Native Hawaiian or Other Pacific Islander; 0 participants chose not to answer.

h Self-reported.

**TABLE 2 |:** Highly valued characteristics of the ICU Care Plan tool.

Theme	Exemplary quotes

Creates a unified, shared frame of reference	*it helps unify what the healthcare team is telling the family or talking to the family about. […] I think if something like this were written out and available not only to families but to the healthcare team, the nurses that actually interact with the family 100 times more than the physician providers do, they can look at what we’re looking at for improvement and look at what we’re looking at for worsening. And so, I think I would use something like this and make it a standard for patients that are truly ICU sick, that we’re expecting to be in the ICU for days. I think it helps the staff have a unified message, which is very helpful*. (ICU physician, participant 30) *If the patient has their designated decision maker, they should also provide it that copy as well, so that they understand what you know, what, what was agreed on and what the plan is. […] So anybody that’s involved, any team that’s involved in caring and responsible for caring for the patient should have a copy of the treatment plan*. (Surrogate, participant 2)
Promotes transparency by illustrating unfavorable outcomes and uncertainty	*I’m glad that they were honest in the parts where they talked about if it didn’t work, that they would make her comfortable. […] And then, being honest and saying that if they took away the breathing machine and it, the medicines weren’t working, she would pass away. So no false hope*. (Older adult, participant 3) *this sort of category of uncertainty, terrible, but here’s what we do know and here’s what we’re going to be looking for and here’s when […] we’re checking in with your family or with you, […] here’s what we’re going to be asking, here’s what we’re looking for. I think that really helps, and it goes a long way to know at least there’s a plan. It feels uncertain and scary but at least somebody has got a plan*. (Surrogate, participant 10)
Sets expectations through its visual layout	*I’m thinking from the point of view of the Hispanic family, I really like the layout of this ICU plan in the way that okay, this is today, because this is what’s going to happen. It goes in, it’s going to go through the plan. And at the same time, it’s going to lay out the outcome for improvement, no change, or worsening*. (Surrogate, participant 15) *it’s just a really good visual descriptor of what the plan is*. (Surrogate, participant 4)

**TABLE 3 |:** Prioritized design features for a tool to support the planning of a time-limited trial.

Theme	Exemplary quotes

*Simplicity…* …in overall design promotes a shared understanding	*this is very simple, it gets right to the point of, okay, is she approving, no changes, and there’s worse conditions that are happening, and possibilities, everything is right there that I can see and think of at this moment for the wishes of the patient, and the family. And this, as a plan, can be set in her chart or with the nurse, the nurse can come on and look and see, okay, this is what the needs are. So, I think it’s a very friendly, useful tool for everybody involved*. (Surrogate, participant 19) *It’s very simple. It does address the main points patients would like to know and physicians would also like you to know […] so patients and I will have very similar lay of land to talk about, we can both see this*. (ICU physician, participant 24)
…and clarity in language prevents ambiguous communication	*I mean, like what is it? An eighth grade reading level you should use? Digestible words. But, you know, that they’re understanding of what’s happening and you know, their input on how to best care for their loved one is important as well*. (Surrogate, participant 4) *I feel like hope is a very tricky word sometimes in the ICU. We often talk—like, if [the patient] ends up in the worsening category. This is a generalization from all kinds of patients. They’re like, “Doctor, you’re taking away our hopes.” And so, I don’t know if it’s a good thing to have those hopes written down or not*. (ICU physician, participant 23)
*Flexibility…* …to individualize the template for each patient	*the language that was used was “For you, [patient], this is what we think”. I think that is so critical because sometimes it comes across like this is just the best practice, this is what we’re going to do. […] knowing that at least they’re about me here. I’m not just another person in this, in this place. This plan is, whether it is specifically for me or not I think the way that it is communicated helps a lot there*. (Surrogate, participant 10) *I like it as is. I wouldn’t change much. And there’s room to write around things. If there’s important family or clinical factors you have to add, you could just write it somewhere on the blanks*. (ICU physician, participant 30)
…to convey that the care plan is not binding or overly rigid	*I think overall the care plan itself is, is simple to understand the way it’s broken down and, obviously, within that there’d be room for modifications if there are unexpected places along the way, and that should also be something that can be the initial part of the care plan* (Surrogate, participant 2) *As a caregiver or a family member, I might hear that as we’re giving her 4 days. If she’s not better, then we’re just, you know, it’s done. So I would rather have heard we’re going to start with 4 days. See where we’re at and revisit this and make decisions from there. You know, and maybe that is sort of how they put it. But you hear things differently in the moment you do*. (Surrogate, participant 9)

## Summary

- Key points
  - A time-limited trial is an approach to patient care that helps navigate challenging decisions about the use of life-sustaining therapies in the intensive care unit (ICU).
  - Time-limited trials of critical care require collaboration among clinicians, patients, and/or surrogate decision makers, yet there is little practical guidance on how to accomplish this and wide variation in current clinical practice.
  - In this qualitative study framed by the human-centered Design Thinking process, we partnered with older adults, experienced surrogates, and ICU physicians to develop a novel tool to facilitate collaborative TLT planning conversations: the *ICU Care Plan*.
- Why does this paper matter?
  - Through a Design Thinking process, we developed a tool to support older adults with critical illness, surrogates, and clinicians as they navigate challenging decisions about the use of life-sustaining therapies.
  - The *ICU Care Plan* tool has promise to help improve care for older adults, as it was universally endorsed by our study participants and described as creating a unified frame of reference, promoting transparency, and setting and managing expectations in such a challenging scenario.
