# Supplementary material for: The ICU Care Plan: Human‐Centered Design of a Tool to Support Time‐Limited Trials for Older Adults With Critical Illness
Source: J Am Geriatr Soc. 2025 Oct 13;73(12):3747–56. doi: 10.1111/jgs.70155 (PMC12650797; doi:10.1111/jgs.70155)
Supplement: Supplementary file 1 — Text S1: Round 1 interviewer/moderator guide. Text S2: Round 2 interviewer/moderator guide. Text S3: Round 3 interviewer/moderator guide. Text S4: Hypothetical patient case used to introduce participants to the concept of a time‐limited trial. Figure S1: Examples of iterative prototypes developed and refined throughout the Design Thinking process. [file JGS-73-3747-s001.docx]

**SUPPLEMENTARY MATERIALS**

**Supplementary Text S1:** Round 1 interviewer / moderator guide

[Introduction script; remind participant about the story book sent via snail mail/email, ask them to gather it and reference it if possible]

I would like to start by telling you the story of a woman named Carol James. This story isn’t about a real patient, but it does describe some experiences that many patients have in an ICU.

[Begin story slides with script, STOP AT SLIDE 11].

I would like to stop here and ask you some questions.

**Domain 1: Building a shared understanding of the time-limited trial care model**

1. I’d like to start by just asking what you think about Carol’s story so far.

*Probes:*

*Have you or anyone you know had an ICU stay like Carol?*

*Have you or anyone you know had to make decisions in an ICU, like Carol and her family?*

*What stood out to you about Carol’s story? Why did this stand out?*

*Was anything surprising to you about Carol’s story? Why was this surprising?*

OK, now I would like to tell you more about Carol’s story. [CONT AT SLIDE 12]

2. What do you think, now, about Carol’s story?

*Probe: What do you think about the second time Carol was in the ICU?*

1. In your own words, how would you describe the new plan for ICU care that was part of Carol’s story?

*Probes:*

*What questions do you have about this new kind of plan for ICU care?*

*How easy is it for you to understand this plan?*

*What parts of this plan are harder to understand?*

**Domain 2: Eliciting needs for successful time-limited trials**

If you can, I’d like you to imagine someone you know well, like a relative, who is in an ICU scenario like Carol. Remember you can look at your story packet, or we can go back and look at the slides, if that helps you.

1. If someone you know well is in a situation like Carol, what would you think about using this new plan for ICU care?

*Probes:*

*What would you need to know to help you think about using this new plan?*

*What would go into your decision when you are thinking about using this new plan?*

1. What kind of support would you need as you were thinking about using this new plan?

*Probes:*

*How could the medical team help you think about this new plan for ICU care?*

*What do you think about receiving information about the plan on a piece of paper?*

*What would you think of receiving information electronically, like through a cell phone app or email?*

**Domain 3: Defining potential benefits and problems**

1. How do you think this new kind of plan can help patients in the ICU and their families?

*Probe: Why do you think someone might choose this kind of plan?*

1. What worries you about this new kind of plan for ICU care?

*Probe: Why do you think someone might choose not to use this kind of plan?*

[Closing script]

**Supplementary Text S2:** Round 2 interviewer / moderator guide

[Introduction script]

**Setting the Stage**

Now, we would like to set the stage for you. We want to re-introduce you to Mrs. Carol James. Carol is a fictional 78-year-old woman living in Wisconsin. She sometimes has trouble breathing because of a problem with her heart. One day while Carol is at home, she starts to have a lot of trouble breathing. She calls 9-1-1 and is brought to the hospital where she is admitted to the intensive care unit (ICU). Carol’s son comes to the hospital right away. The medical team is worried about Carol’s breathing and wants to talk with her and her son about using a breathing machine to help her. They are not sure if Carol’s breathing will improve or worsen during this time. Carol’s medical team sits down with Carol and her son to discuss the treatment plan for her ICU care. The medical team uses a tool to help guide the conversation and come up with Carol’s treatment plan.

Today we want to hear your thoughts and opinions about new tools that could be used during such a conversation [distribute physical copies and start slide sharing; remind participants about tool examples sent ahead of time].

We are going to give you a few minutes to look at Tool #1 and Tool #2 and write down any thoughts, comments, and questions you have about them. When we come back together, we will ask each of you to share your initial reactions to the tools.

**Initial reactions**

After 3-5 minutes (take note of when participants are done writing), go around and ask each person:

- What are your initial reactions to these tools?
  - In 1-2 words, what comes to mind?
- How would you describe this piece of paper when talking with others?
  - We have been calling it a tool. Are there other descriptions/words that you would use?

Thank you so much for sharing your initial reactions to the tools.

[The following categories will be covered during the focus group, but the order may differ depending on what participants share initially. For example, if participants focus mainly on the visual layout of the tools, the facilitator will start with those questions and come back to the language-related questions.]

**Language**

- What are your thoughts about these words/phrases? [address each one individually]
  - Looking for
  - Next steps
  - Our ICU plan
  - Hoping for/fearful of

[If there is confusion about hoping for/fearful of, clarify that we included this based on feedback from the first interviews in which participants said what is most important to the patient should be prioritized.]

- *Probes*
  - *What kind of information would you expect to see in each box?*
  - *Are there any other words you would prefer instead?*

**Visual Layout**

- What surprised you about the tools?
- What are your thoughts on the placement of each box?
- What do the dotted lines mean to you?
  - For example, around “meet again on,” “possible next steps,” and “improving/no change/worsening”

**Tool Use and Sharing**

- What is helpful about the tool?
- What is missing from the tool?
- What is too much/could be taken out from the tool?
- Would it be helpful to have anything on the back of the tool? If so, what would you want to see in that space?
- How do you see the tool being used with patients/families?
  - How might this tool change care for patients/families?
- How might the tool be shared with the rest of the healthcare team?
  - Picture in healthcare record? Copy in patient’s room?

**Closing**

We have discussed so much today. To close we would like to ask one final question:

*What is the #1 most important thing for us to focus on as we consider revising the tool?*

[Closing script]

**Supplementary Text S3:** Round 3 interviewer / moderator guide

[As participants come onto Zoom, remind them about the tool sent to them in advance (via email) and ask them to locate it as reference, if possible. This can be done verbally and reminder put in the chat.]

[Introduction script]

**Setting the Stage [start slide share / story book ]**

Now, we would like to set the stage for you. We want to introduce you to Mrs. Carol James. Carol is a fictional 78-year-old woman living in Wisconsin. She has severe heart failure which has been complicated by recurrent exacerbations and contributes to chronic shortness of breath. Carol is now admitted to the ICU with another heart failure exacerbation and her breathing is worsening to the point that you are considering whether to pursue intubation and mechanical ventilation. She also has an acute kidney injury and she is not responding quickly to IV diuretics. She does not currently have decision making capacity, so you are planning to meet with Carol’s son to talk about what this means and what to do next. Carol has previously told her family that she hopes to live as long as possible but that she would not want to be dependent on life support for a long time.

For the purposes of our conversation today, you are Carol’s ICU physician. Also, based on the clinical information at this point, you are uncertain as to whether Carol will improve over the next several days or not.

We have designed this tool to help with conversations like this one, with Carol’s son. The idea is to use this tool as a template that would be filled out with patients or their families, together during a conversation. The tool is designed to be printed on paper and filled out in the moment, and family members can keep the copy at the end of the conversation. Today, we want to hear your thoughts and opinions about the tool that would be used during such a conversation.

**Showing the tool + initial reactions**

Now we’re going to share the tool on the screen.

We are going to give you a couple of minutes to think about how you would use this tool in your conversation with Carol’s son. We’d like you to try fill out the grey spaces on the tool, considering what you might write in these spaces when having a conversation about a patient like Carol. Please feel free to conjure up your own details or specificity for this clinical situation. There are no right or wrong answers.

I’m going to give you 3 minutes to think about this

*[After 1-2 minutes (take note of when participants are done writing) ask:]*

- What are your initial reactions to this tool?
  - What was the first thing that you noticed?
  - Where did you get stuck or confused?
  - Did it raise any concerns for you?
    - What are they? (And why?)

Thank you so much for sharing your initial reactions. Now we’d like to move onto some more specific questions and ask more about what you wrote in the tool, building on what you’ve said already.

[The following categories will be covered during the interview, but the order may differ depending on what participants share initially. For example, if participants focus mainly on the visual layout of the tools, the facilitator will start with those questions and come back to the language-related questions.]

**Content**

- I’d like to ask you how you filled in different parts of the tool. [address each one individually]
  - Try life support for _______ days
    - What did you write?
    - How did you decide on that number of days?
      - In general, how might you decide what to write in here?
    - What concerns come up for you as you fill this out?
    - Was it easy to set the number of days?
    - What do you think about the wording of this part of the tool?
  - Signs to look for during this time
    - What did you write in these boxes?
    - How did you decide what to write?
    - What concerns come up for you as you fill this out?
    - Was it easy to think of what to write?
    - [If not easy] What could make it easier?
  - Hopes, Fears, What they Enjoy
    - What, if anything, did you write in this box.
    - What do you think about the words used on this box?
  - Are there other words or phrases on the tool that you’d like to discuss?
  - *What was your first thought/what questions came to mind as you fill this out?*

**Visual Layout**

- What are your thoughts on the placement of each box?
- Is there anything you’d like to see added to the tool?
- Is there anything that could be taken out from the tool?
- We’ve intentionally left space under the date of the next steps so that physicians (if they wanted to) could describe the next steps. The steps might be continuing recovery-directed or comfort focused care. What do you think about having this space?
  - What would you think if there was instead a box that said “potential next steps?”

**Tool Use and Sharing**

- When or for whom would you consider using this tool?
  - Are there specific types of clinical situations that you wouldn’t use this tool for?

For the moment, let’s go back to Carol’s case. I’d like you to imagine that right now you are using this tool and speaking with Carol’s son during a family meeting.

- Walk us through how you might use this tool with Carol’s son
  - How would you introduce this tool in the room?
  - Ask about each section?
    - *If they didn’t speak about a certain section ask about why*
    - *Which sections of the tool were hard to talk about?*
- How might the tool be shared with the rest of the healthcare team?
  - Picture in healthcare record? Copy in patient’s room?
- I’ve asked you to now consider using this tool during a family meeting. Would you consider using this tool in any other settings when communicating with patients or families?
  - Telephone, video conference, on rounds, or bedside?
- How might this tool change care for patients/families?

**Closing**

I’d like to end by asking one final question:

*What is the #1 most important thing for us to focus on as we consider improving the tool?*

[Closing script]

**Supplementary Text S4**: Hypothetical patient case used to introduce participants to the concept of a time-limited trial. Study participants were provided with a booklet prior to their initial participation in the study that included the following story board with the associated text. The Round 1 study sessions also included a visual presentation of the case. We also used a modified version of this case in the Round 3 physician study sessions, with clinical details and language directed at a physician audience.

| 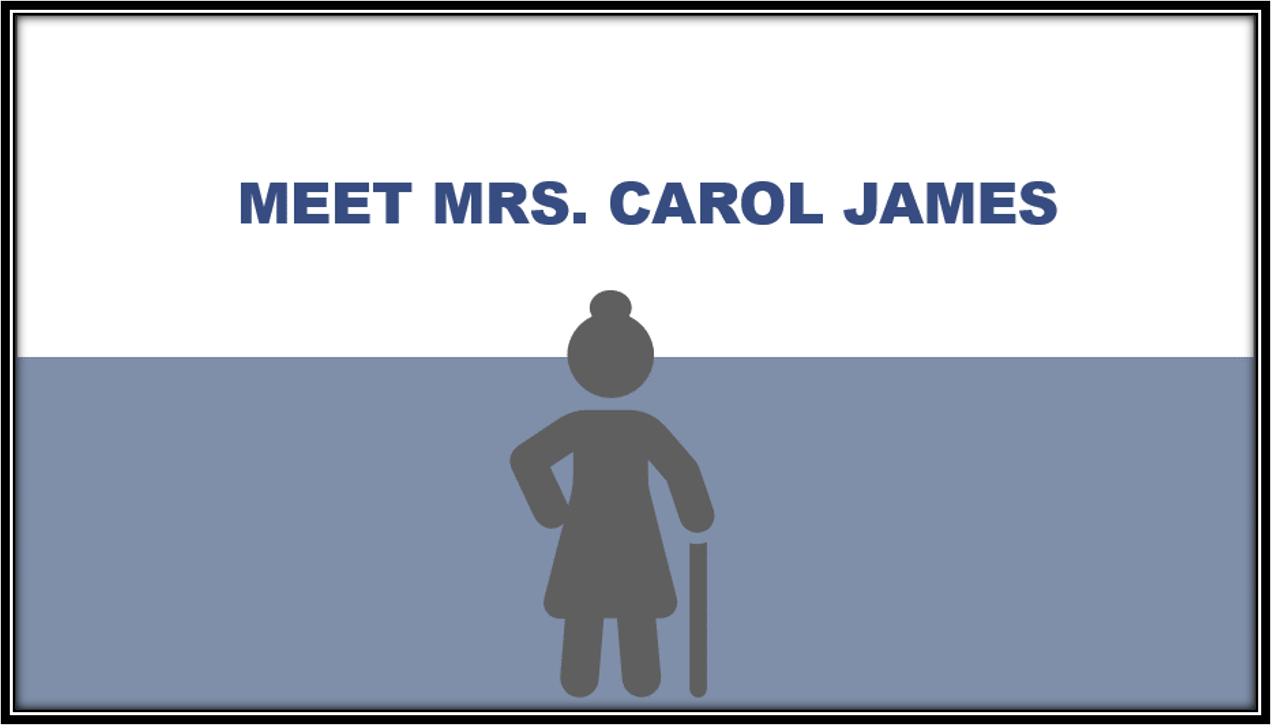 | I would like to start by telling you the story of a woman named Carol James. This story isn’t about a real patient, but does describe experiences that many patients have had in an ICU. |
| --- | --- |
| 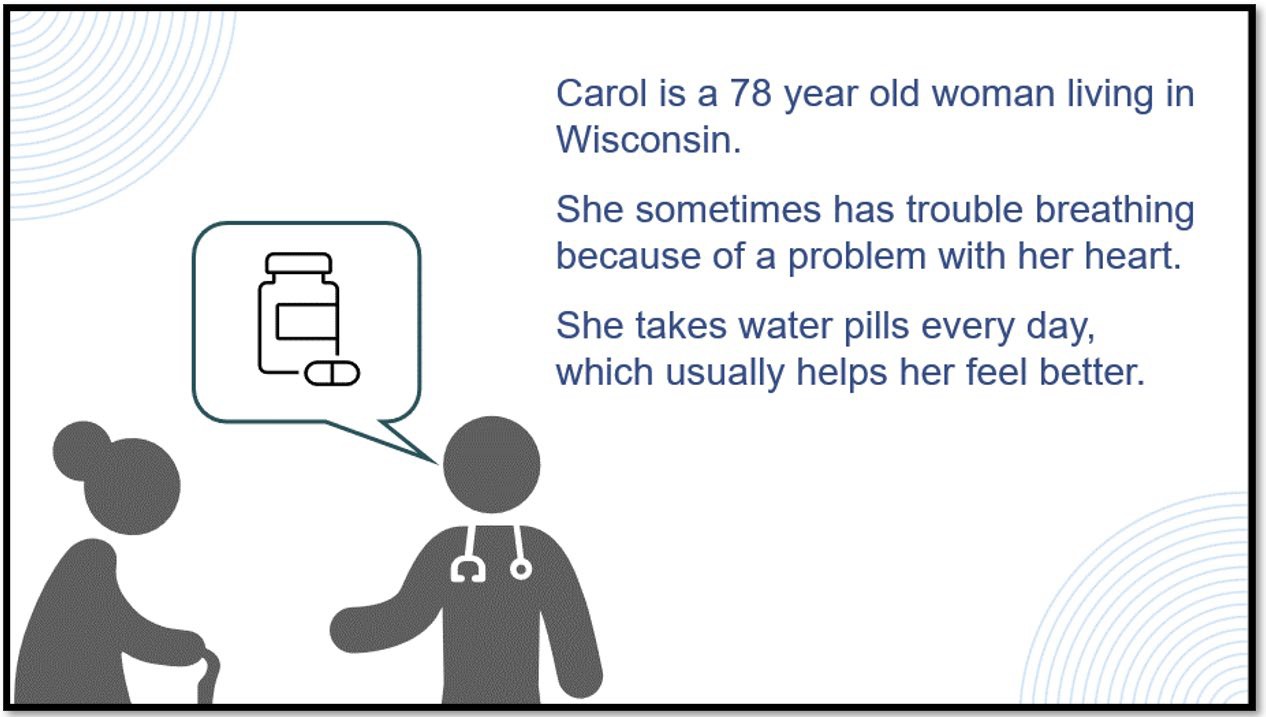 | Carol James is a 78 year old woman living in Wisconsin. She sometimes has trouble breathing because of a problem with her heart—the doctors told her that her heart muscle is stiff. She takes pills every day that help take away the extra water in her lungs. This makes it easier for her to breathe, and usually helps her feel better. |
| 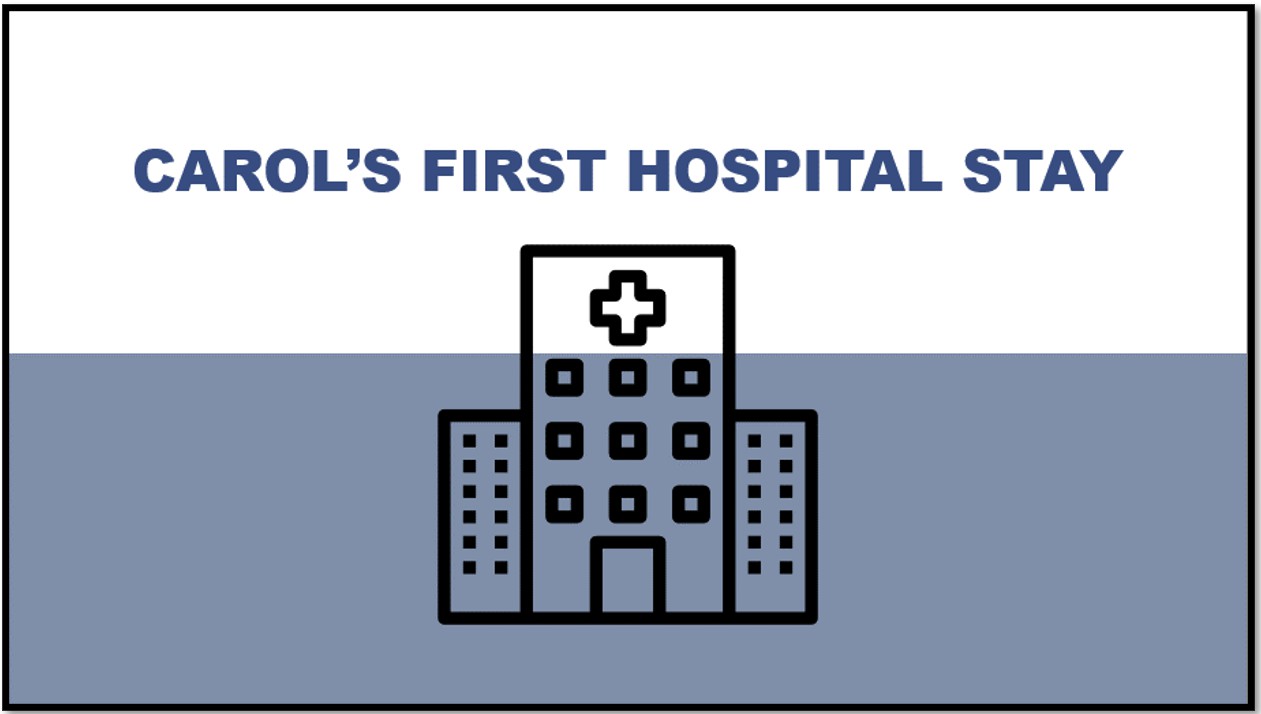 | One day while Carol is at home, she starts to have a lot of trouble breathing. This time, her water pills do not help. |
| 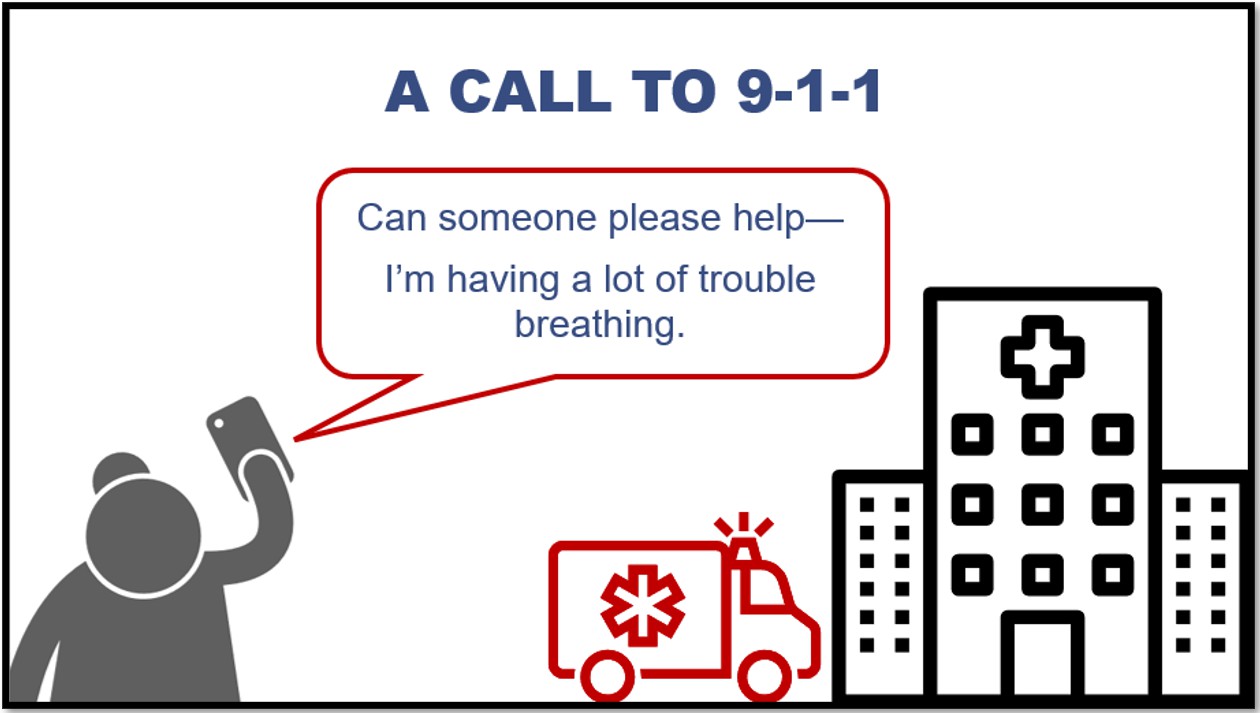 | Carol calls 9-1-1 and is brought to the emergency room where the doctors give her oxygen and other medicines to help her breathe. She is admitted to the ICU, and her family comes to the hospital right away. |

| 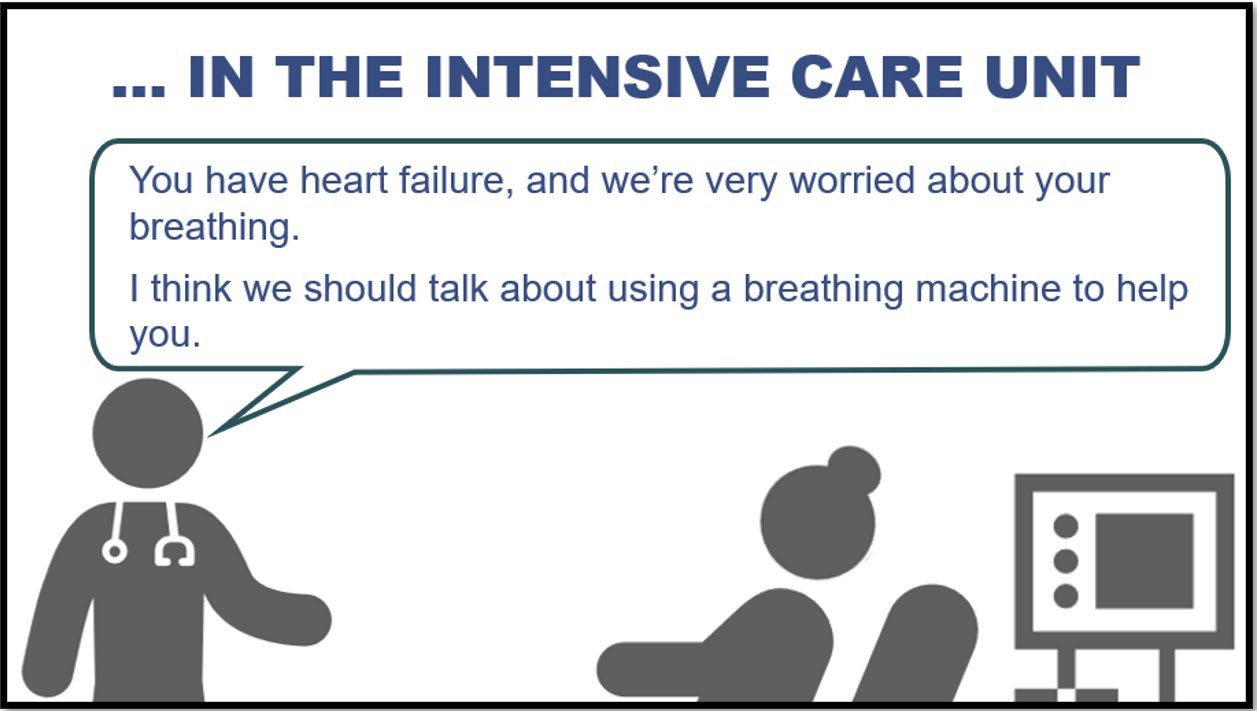 | Since Carol's breathing problems are not getting better quickly, the ICU medical team talks with her about the possibility of using a form of life support called a mechanical ventilator, which is sometimes called a breathing machine. The medical team explains that this machine can help support her breathing, and they would also be giving her medicines to help with the cause of her breathing problems. |
| --- | --- |
| 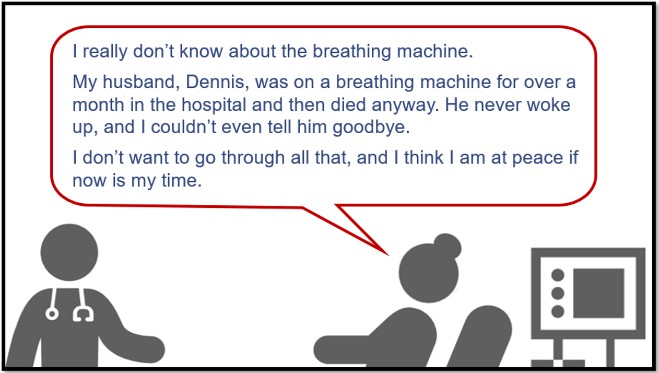 | Carol talks with the ICU medical team about her worries—she really isn’t sure about what she wants to do.  She tells the team about her experience with her husband, Dennis, who went through something similar a few years ago. Dennis had different health issues, but he also went to the ICU with breathing problems. The doctors told him that he needed the breathing machine, but after they started it, he never got better. He went through a lot. He was on a breathing machine for more than a month and then died while on the breathing machine without ever waking up.  Carol reflects on how hard it was to watch Dennis go through that, and how hard it was for their family during that time. She says to the medical team, “I don’t want to go through all that and I don’t want to burden my family with those decisions. I think I am at peace if now is my time.” |
| 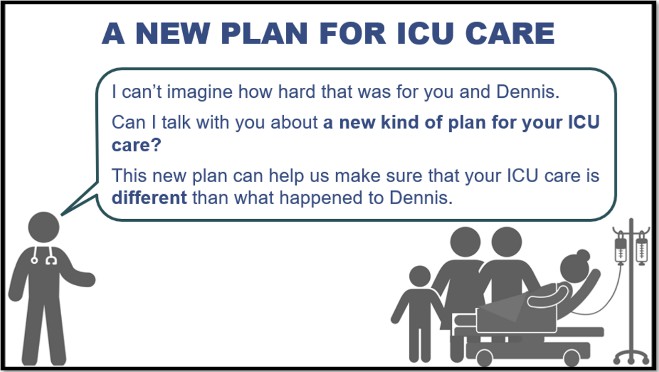 | Hearing Carol’s story about Dennis, an ICU doctor reflects on how hard it must have been for both Carol and her husband.  After some time, the doctor asks Carol if they could talk about a new kind of plan for her ICU care. With this new kind of plan, Carol would be able to guide the decisions that might come up after we start the breathing machine. The doctor thinks this new plan can help make sure that her ICU care experience  is different than what happened to Dennis. |

| 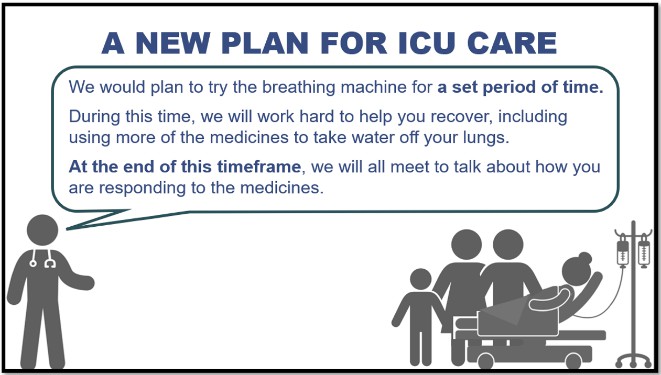 | The doctor suggests the following plan:  We would plan to try the breathing machine for a set period of time. During this time, we will work hard to help you recover, including using more of the medicines to take water off your lungs. For you, Carol, I think we would know if the medicines are working within four days. At the end of this timeframe, we will meet with your family to talk about how you are responding to the medicines. During this meeting, we will revisit your wishes, which you have helped make clear to all of us today. |
| --- | --- |
| 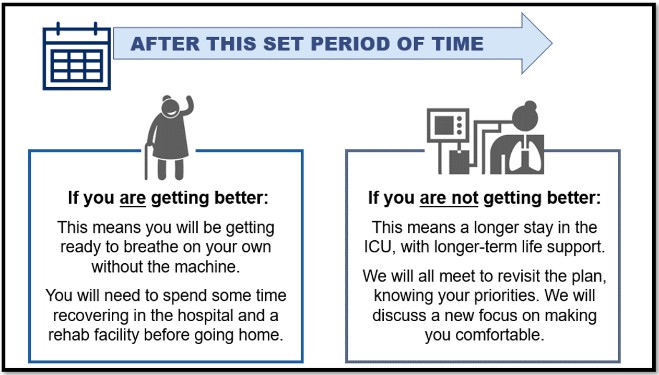 | If the medicines are helping Carol, then after four days she would be getting ready to breathe on her own without the machine. In this case, Carol would likely need to spend some additional time recovering in the hospital and then in a rehabilitation facility, but ultimately, we would hope that she can go home again.  If the medicines are not helping or Carol starts having other problems over the next four days, that means that Carol would have a longer stay in the ICU with long-term life support like her husband Dennis had. At that time, everyone would meet together to discuss. Knowing Carol's wishes and priorities to avoid long-term life support, they could consider a new focus on making Carol as comfortable as possible. This would mean stopping the ventilator, letting Carol die naturally.  After hearing about this new kind of plan for ICU care, Carol and her family ask several questions of the medical team. They talk  through the plan together. |

| 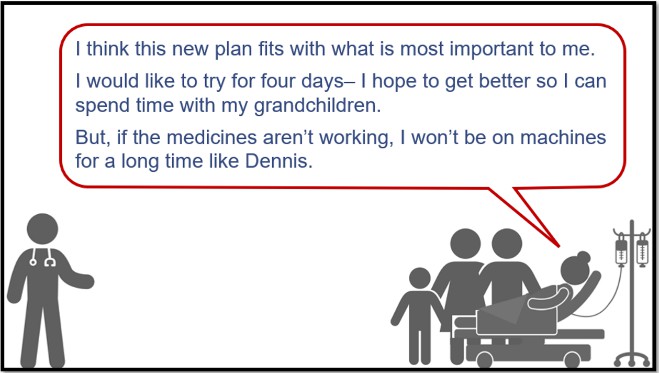 | Carol decides this plan fits with what is most important to her. She is hoping to return home and to enjoy time with her grandchildren if she gets better with several days of life support, but she is also comforted knowing that if the plan isn’t helping, she will not end up with long-term life support like Dennis.  Understanding this, the medical team starts the breathing machine and continues the medications to help with her breathing. |
| --- | --- |
| 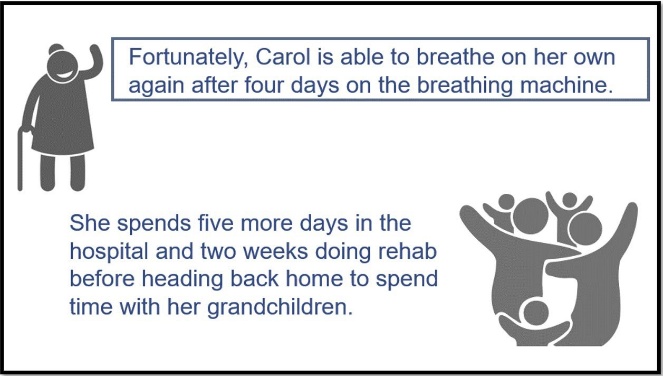 | Fortunately, Carol starts to get better with this care. She is able to breathe on her own without the machine in four days. After a few more days in the hospital and two weeks at a rehabilitation hospital to regain strength and heal, Mrs. James is able to go back home and spend time with her grandchildren. |
| 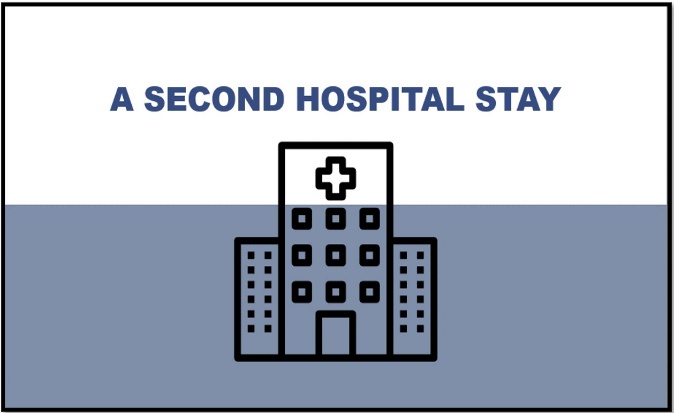 |  |
| 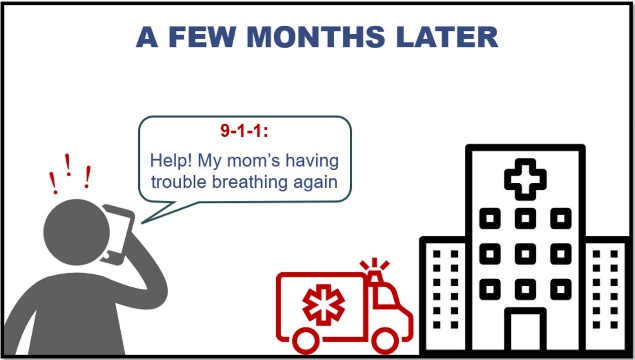 | Several months after her first hospital stay, Carol is having trouble breathing again and comes back to the ICU, after her son calls 9-1-1. |


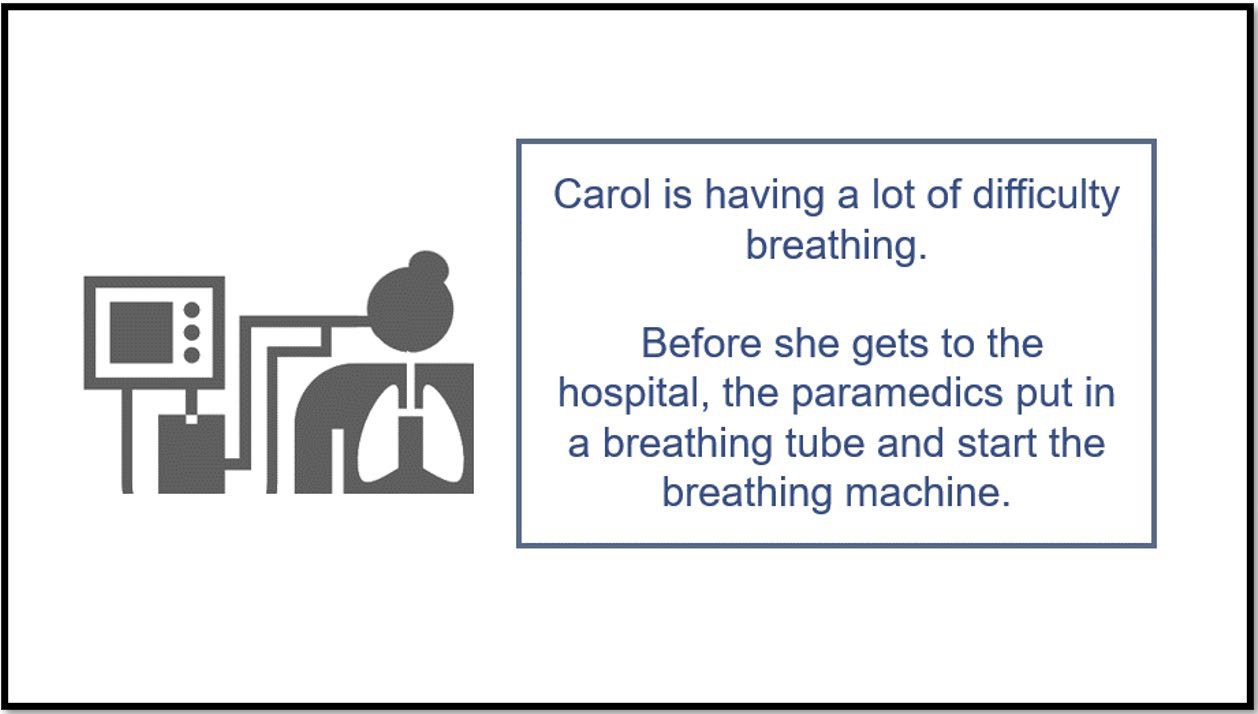
Carol is having such a hard time breathing at home, the paramedics put in a breathing tube and start the breathing machine before she even gets to the hospital.


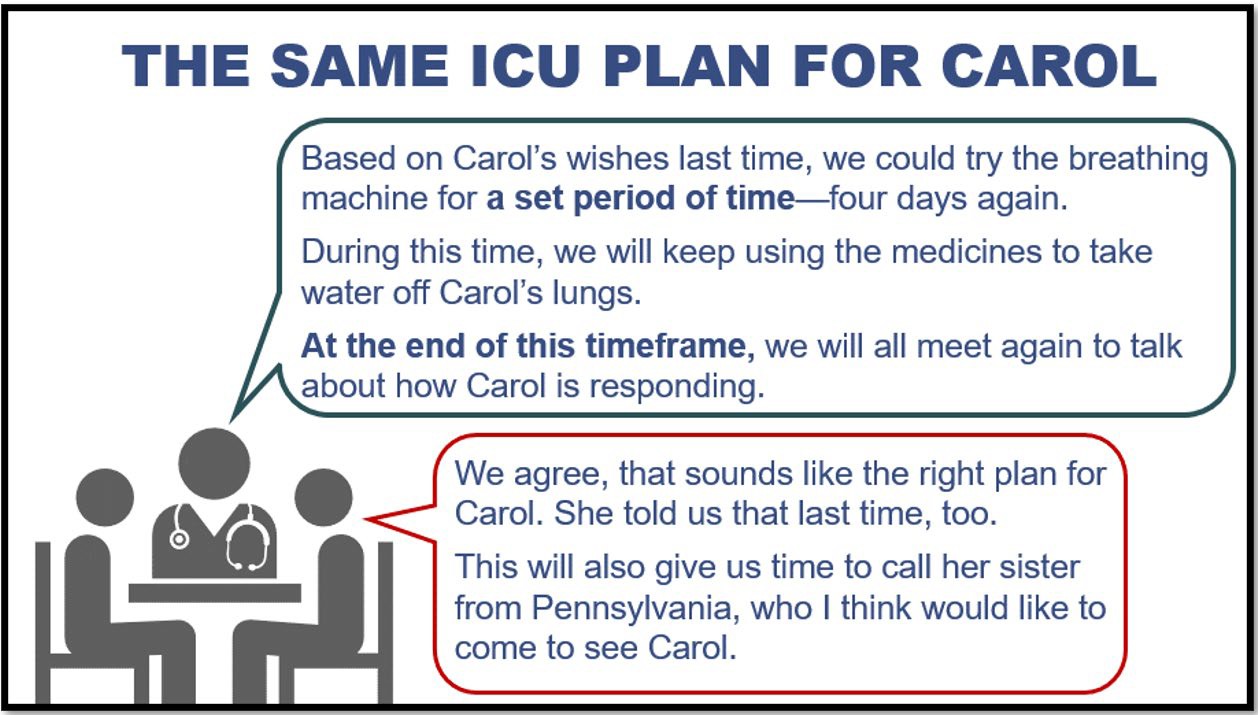
The medical team speaks to Carol’s family about trying the breathing machine for a set amount of time, just like the last time she was in the ICU. The doctors and her family talk about what Carol told them last time, about what was most important to her for a situation like this. The doctor thinks four days will be the right amount of time to find out if the medicines and breathing machine are helping Carol.


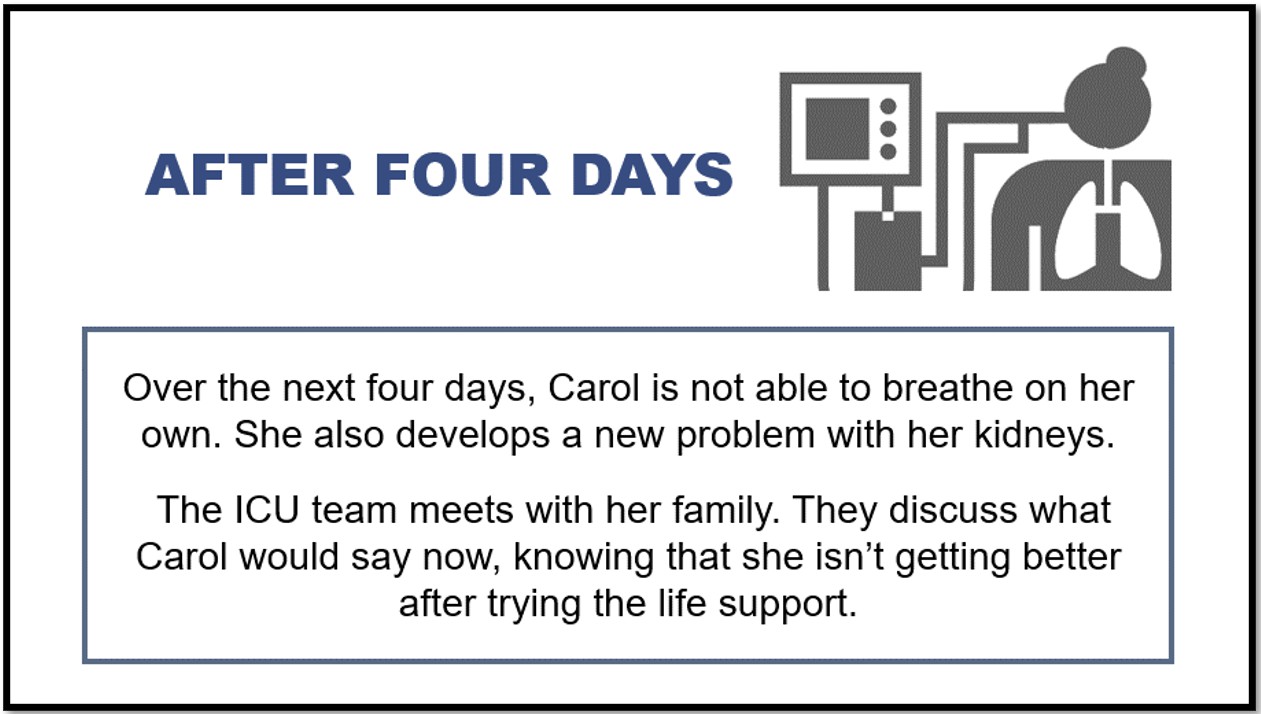
Over the next couple of days, Mrs. James unfortunately needs more and more support from the breathing machine. She also starts to have a new problem with her kidneys. She is receiving medicines to treat the discomfort caused by the breathing tube and machine, so she is not awake most of the time.

After four days, the medical team meets with her whole family. They discuss the tests and signs that they were using to measure Carol’s progress, and they describe the possible outcomes and care that she might need if they were to continue with the breathing machine. They also spoke to the family about the option of stopping the breathing machine. This means that Carol would probably pass away soon after. If they chose this option, their focus would be on making sure Carol is as comfortable as possible knowing that Mrs. James did not want to be kept alive on machines if she wasn’t getting better.


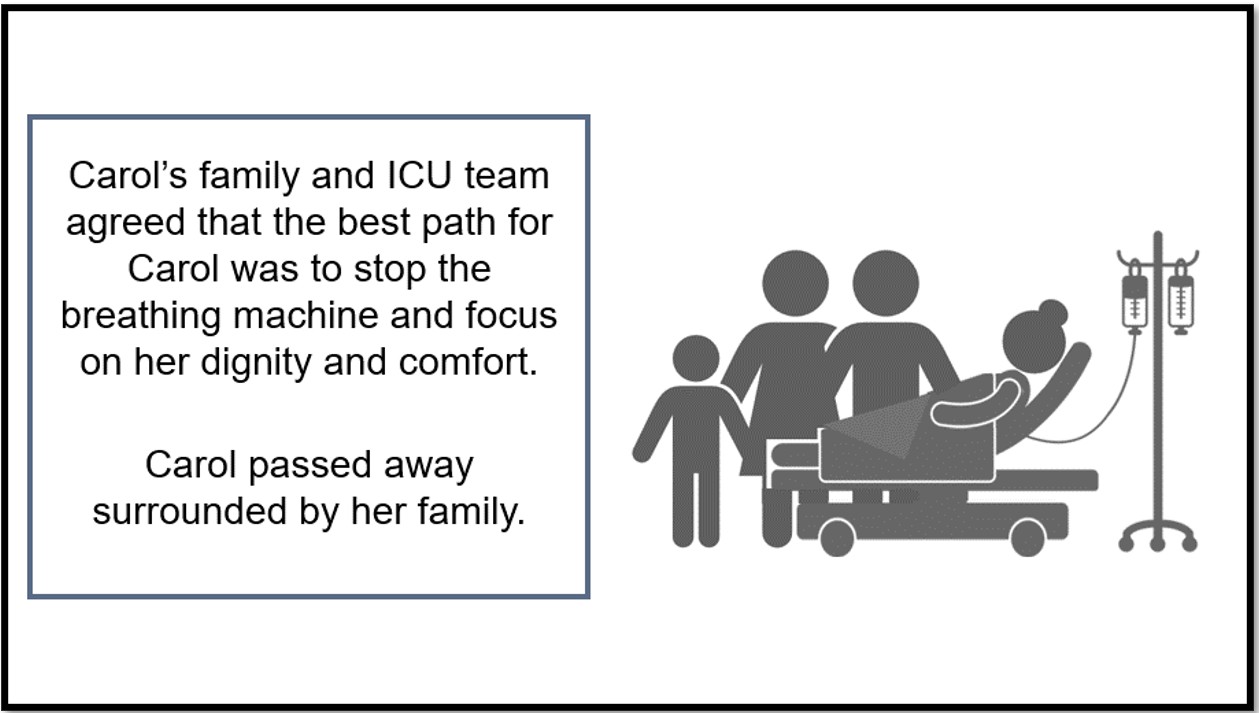
Knowing what is most important to Carol, the medical team and Carol’s son make the decision to stop the breathing machine and focus on Carol’s wishes, and her dignity and comfort. She passed away surrounded by her family and friends at her bedside.


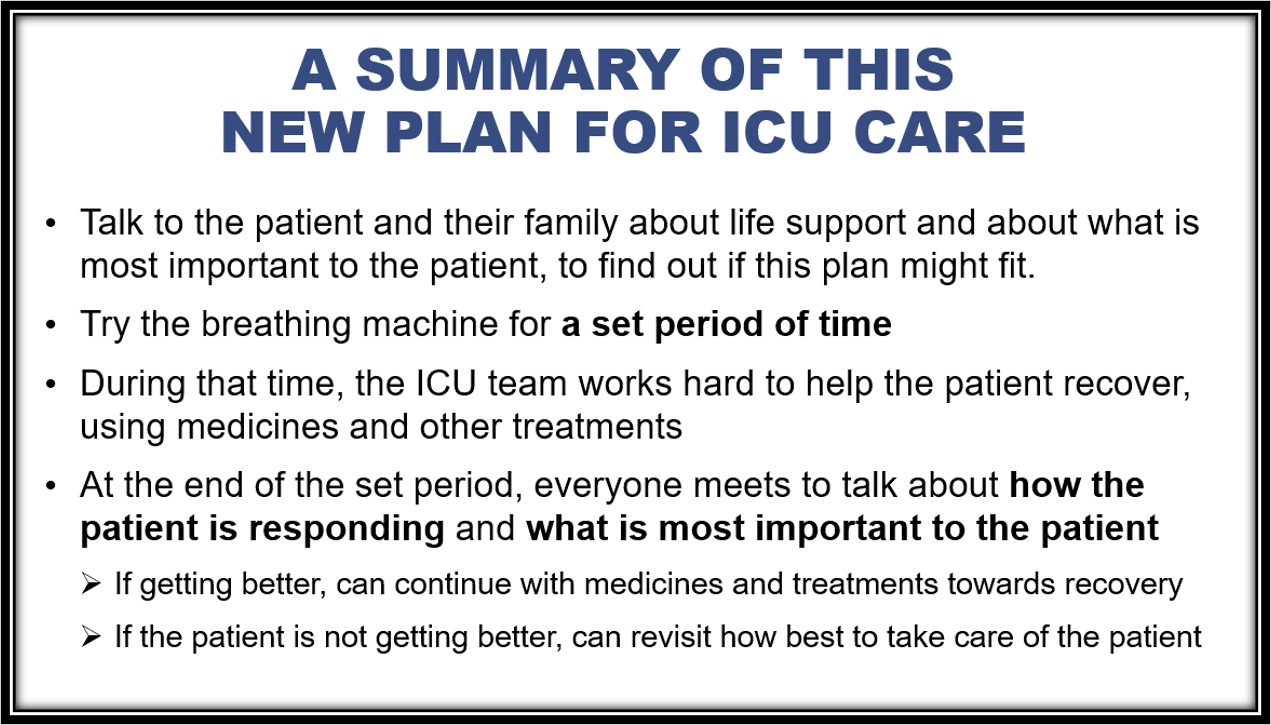


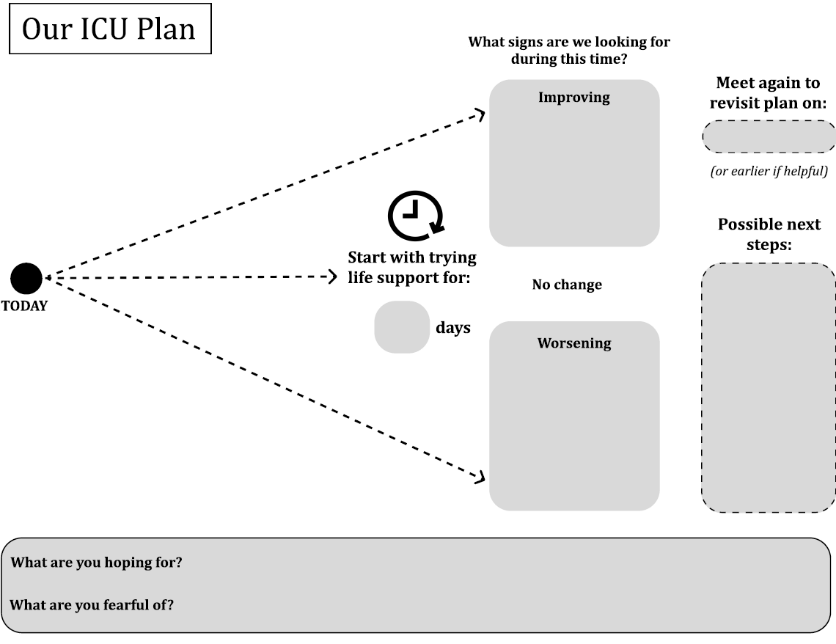

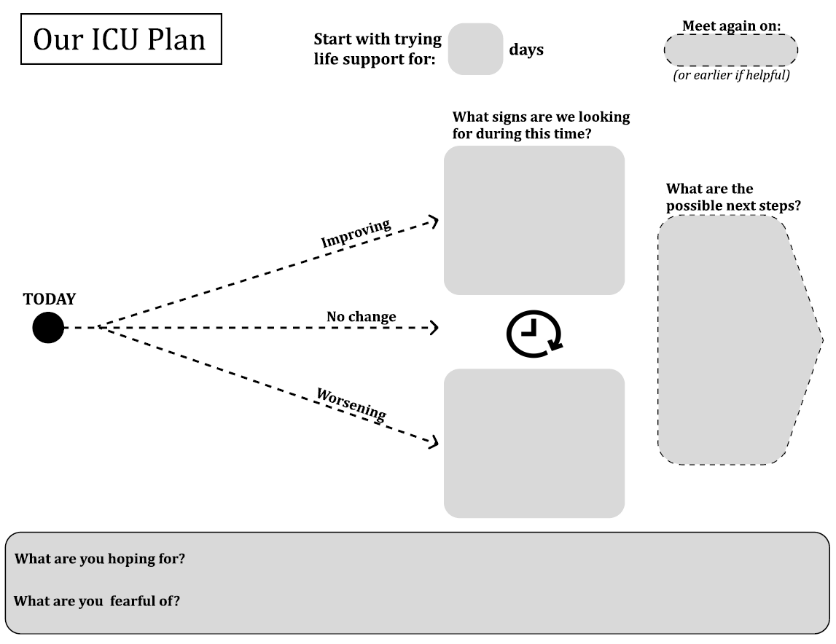


**
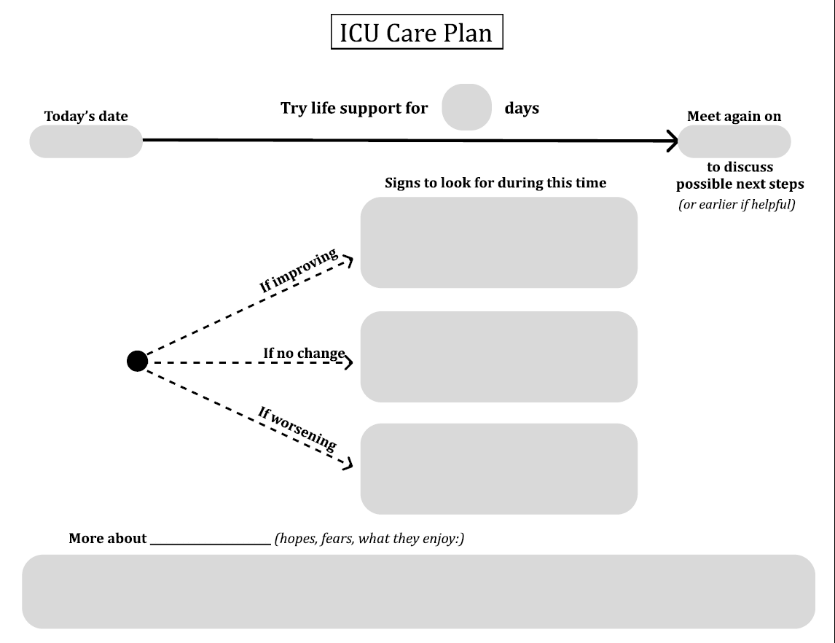

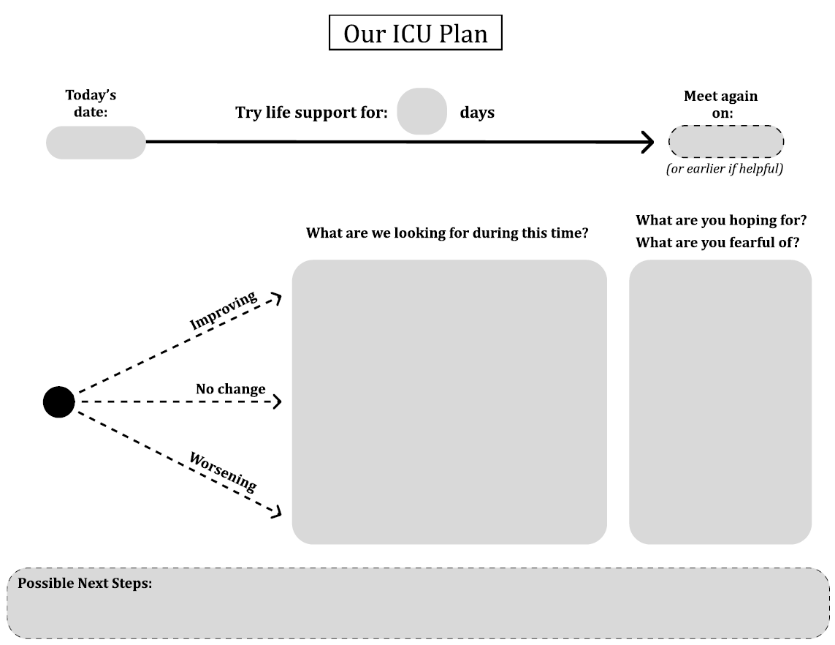
**

**Supplementary Figure S1: Examples of iterative prototypes developed and refined throughout the Design Thinking process.**
